# Reduction of an Unusual Salter-Harris Type IV Fracture of the Ulna

**DOI:** 10.1155/2020/8498401

**Published:** 2020-03-03

**Authors:** Stephanie Marrannes, Delphine Lambrecht, Arne Decramer

**Affiliations:** ^1^Department of Orthopaedic Surgery and Traumatology, Ghent University Hospital, Belgium; ^2^Department of Orthopaedic and Trauma Surgery, AZ Delta Hospital, Belgium

## Abstract

We report the case of a 14-year-old boy with an isolated Salter-Harris type IV physeal fracture of the distal ulna. Following failed closed reduction, transition to open reduction and pin fixation was required. Six-month follow-up showed a favourable clinical evolution. Evidence suggests that long-term follow-up is needed because of the increased risk of premature physeal closure and subsequent growth disturbances associated with this type of injury.

## 1. Introduction

Fractures of the distal forearm are common in children and mostly involve the radius. Isolated fractures of the distal ulna are rare. Fractures involving the ulnar physis only account for approximately 4% of all physeal injuries [1–4]. Treatment consists of anatomic reduction to maximize chances for continued growth [5]. Closed reduction is often unsuccessful due to soft tissue interposition, so this type of fracture is generally stabilized with open reduction and fixation [6–9]. Growth disturbances due to premature closure of the physeal plate following fracture of the distal ulna are frequent [10]. The distal ulnar physis accounts for 70-80% of the ulnar growth. Depending on the age at the time of physeal closure, growth arrest can lead to significant ulnar shortening with compensatory changes in the radial development [6].

## 2. Case Report

**Figure 1:**
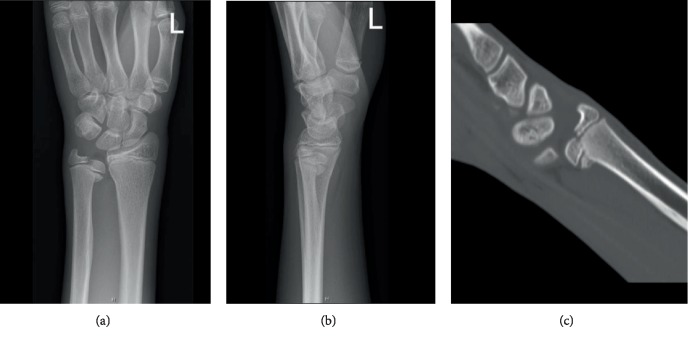
(a, b) Anteroposterior and lateral X-ray after trauma showing an isolated Salter-Harris type IV fracture of the ulna. (c) Sagittal CT image showing an intraarticular step of 3 mm.

A 14-year-old boy presented to the emergency department with a painful left wrist following a fall with his bike after a low-velocity collision with a car. The exact mechanism of trauma could not be described by the boy, but most of the impact was received on the left hand and wrist. On clinical examination, there was swelling but no gross deformity of the left wrist. The wrist was diffusely tender on palpation and range of motion (ROM) was limited by pain. There were no neurovascular deficits and the skin was intact. Anteroposterior and lateral X-rays of the left wrist showed a displaced Salter-Harris type IV fracture of the distal ulna (Figures 1(a) and 1(b)). Because of this uncommon fracture type with displacement of the fragment, subsequent computed tomography (CT) was performed. CT showed a displaced fracture through the epiphysis and metaphysis on the volar side of the distal ulna with an intraarticular step of 3 mm (Figure 1(c)). The patient's wrist was immobilized in a below-the-elbow plaster at the emergency department. Reduction and stabilization were, for practical reasons, planned 5 days later.

**Figure 2:**
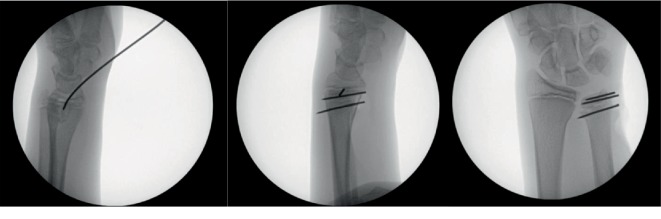
Radioscopic control of pin placement during surgery.

The procedure was performed under general anaesthesia. First, an attempt at closed reduction was made, with pressure applied to the volar aspect of the wrist under radioscopic control. This resulted in a partial but insufficient reduction of the fracture, which led to conversion to an open reduction and fixation procedure. The fracture was approached through an ulnovolar incision. The fragment was reduced under radioscopic control using Kirschner pins to manipulate and lever the fracture fragments. Three Kirschner pins were placed parallel to the physeal plate. Two pins were placed in the coronal plane with one through the proximal fragment and one through the distal fragment. A third pin was drilled in the anteroposterior plane through the distal fragment (Figure 2). The patient was immobilized in an above-the-elbow cast for two weeks.

**Figure 3:**
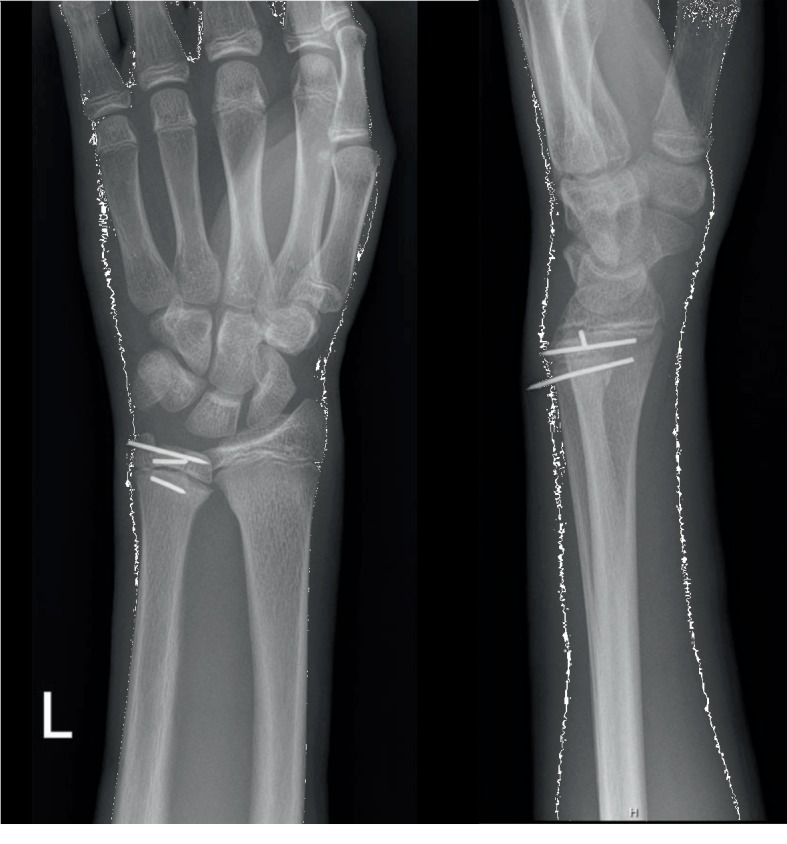
Anteroposterior and lateral X-ray 2 weeks after surgery.

Follow-up after two weeks showed a favourable clinical evolution with maintained position of the fracture fragments on X-ray (Figure 3). To encourage mobilization of elbow and fingers, a wrist brace had to be worn for three more weeks. Rotations had to be avoided. One K wire was removed after six weeks and automobilization was started. Evaluation four weeks later showed a good range of motion in flexion-extension and rotations of 45°. To improve supination, physiotherapy was added. Three months later, the second pin was removed because of migration.

**Figure 4:**
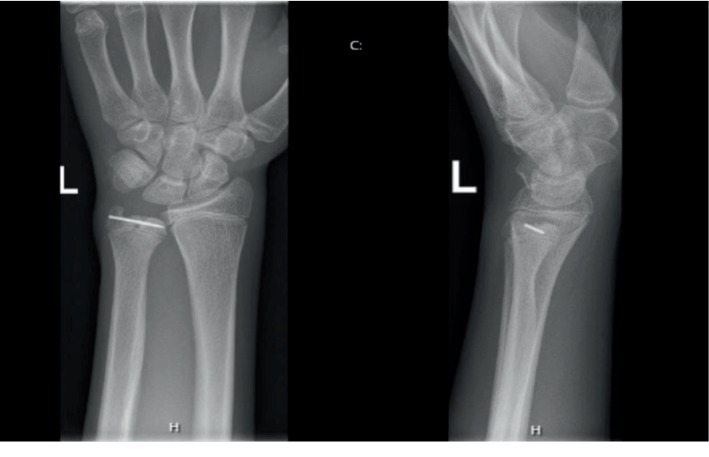
Anteroposterior and lateral X-ray 6 months after surgery. Two pins have been removed. No physeal closure visible.

**Figure 5:**
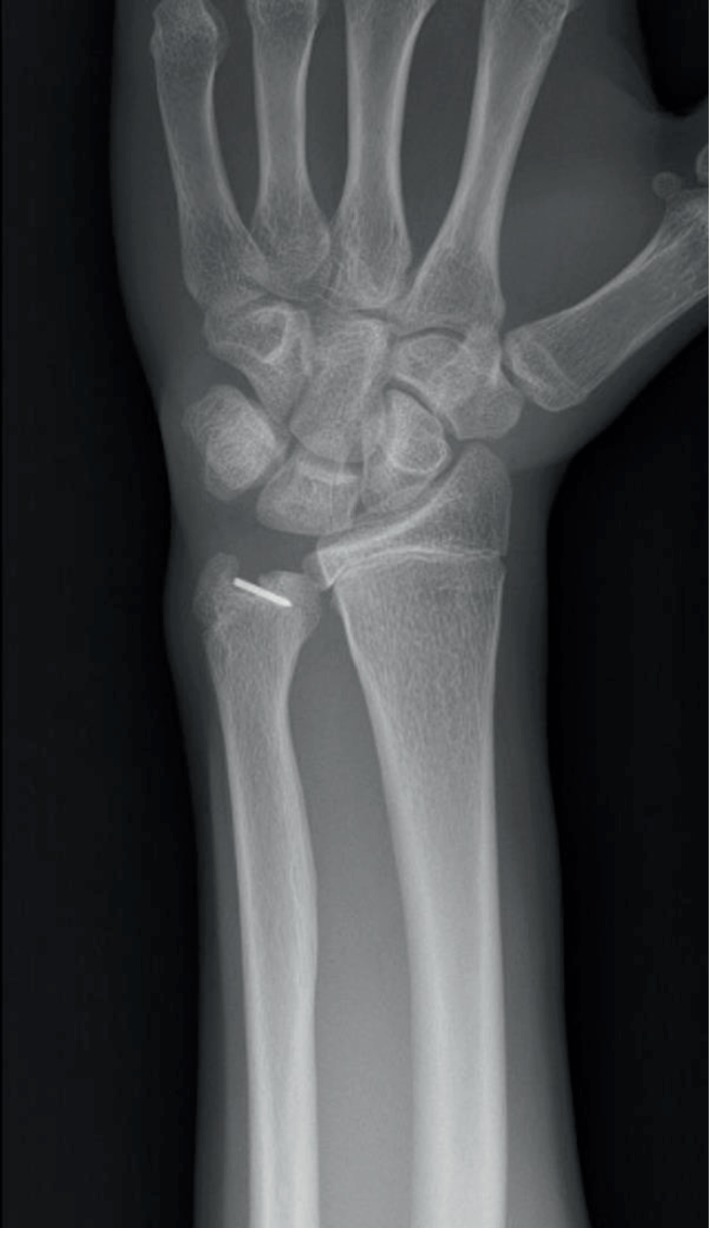
Anterioposterior and lateral X-ray 1 year after surgery shows premature physeal closure and mild negative ulnar variance.

At follow-up 6 months after injury, there was a full range of motion of the wrist compared to the other side with a similar grip strength on both sides. X-ray of the wrist showed the fracture was fully healed with no evidence for premature fusion of the physis or growth arrest (Figure 4). X-ray 1 year postoperatively showed complete closure of the physis with development of a negative ulnar variance (Figure 5). Further follow-up with radiographic control will be provided until skeletal maturity.

## 3. Discussion

Fractures of the forearm are the most common (45%) fractures in children [1]. In most cases, the distal radius is injured; fractures of the distal radius comprise 20-35% of all paediatric fractures. One-third of all radial fractures involve the radial physis [2]. In comparison, injury of the ulna is rare. Reports suggest that physeal fractures of the ulna only account for ±4% of all physeal injuries [3, 4]. Fractures of the ulnar growth plate are usually associated with fractures of the distal radius. Isolated injuries of the distal ulna are infrequent, which could possibly be explained by the anatomy of the ulnocarpal joint where the TFCC provides a cushioning effect for traumatic forces applied to the wrist [1, 6, 7, 10]. Premature physeal closure (PPC) with subsequent growth disturbance is a well-known complication following physeal injury [1, 4, 10].

The most common type of ulnar physeal fractures are Salter-Harris (SH) type II fractures [2]. There is limited literature available about SH type IV fractures of the ulna. Our search of the current literature conducted in MEDLINE, Embase, and Web Of Science yielded only 6 elaborated case reports about SH type IV ulnar fractures and some brief notes about this type of fracture in review articles about physeal injury of the forearm [1, 6–10]. Of these 6 articles, only one described an isolated ulnar fracture without radial involvement [7].

The exact mechanism of trauma producing this atypical fracture pattern is unclear. All six articles on SH type IV ulnar fracture report falls on an outstretched hand as the cause of injury. However, these were mostly combined ulnar and radial fractures. Isolated fractures of the distal ulna are often a result of direct impacts to the ulna [11–14]. Nelson et al. suggested that type III and IV fractures of the ulna are rarely caused without either extreme ulnar deviation or displaced radial fractures [15]. Hinohara proposed that isolated physeal injury of the distal ulna could also be caused by a forceful, combined ulnar deviation-dorsiflexion of the wrist in a pronated forearm [16].

In all six cases reporting on SH type IV fractures of the ulna, reduction was achieved through an open reduction and fixation using K wires after a failed initial attempt at closed reduction. Four of the articles described interposition of soft tissue between the fracture fragments, making closed reduction impossible. Interposition of the extensor carpi ulnaris, joint capsule, and periosteum has been described [6–9, 17]. Repeated attempts of reduction and pinning increase chances of PPC [1, 4]. Therefore, we advise keeping a low threshold for conversion to an open procedure, performing only one attempt at closed reduction [4]. Open reduction and fixation should pursue complete anatomic reduction, minimizing articular disruption of the distal radioulnar joint (DRUJ) and distal ulnar articular surface [5]. To achieve this, Kasis et al. and Faraj et al. placed their wires in a similar manner as we did, with 3 wires parallel to the growth plate [7, 8]. O'Hagan et al. placed two K wires vertically through the fragment thus piercing the growth plate [6]. Immobilization was mostly done using a long arm cast for a duration of 6-8 weeks [6–8]. We had no arguments to cause displacement of fracture fragments with conversion to wrist brace immobilization after two weeks to encourage mobilization of the elbow and fingers.

Despite all articles in this review using the Salter-Harris classification to describe these fractures involving the physeal plate, some authors state that the pattern of physeal injury is not significantly correlated with the clinical outcome of these injuries. It is suggested that age at trauma, high-energy trauma or open fracture, and repeated attempts at reduction are better prognostic factors [1, 10]. Regarding PPC, Golz et al. report an incidence of 55% following ulnar physeal fracture [10]. Most of the articles included in this overview describe at least a partial premature fusion of the physis. The duration of follow-up required to identify PPC is unclear. A known consequence of premature fusion of the physeal plate is axial shortening of the bone. The distal physis of the ulna accounts for 70-80% of the ulnar growth. Ulnar shortening can lead to compensatory mechanisms in the radius such as radial bowing, increased ulnar angulation of the radial articular surface (presumably caused by a tethering effect of the intact TFCC), ulnar translation of the carpus, and instability of the DRUJ. This can cause persistent pain, limited range of motion—most present in pronation and radial deviation—and loss of grip force. Negative ulnar variances ranging from 0.8 to 4 cm were reported in the included articles. One article mentioned visible growth plate disturbance six months after injury [8], which was not a problem in our case.

Notably, the ulnar shortening is often cosmetically displeasing for patients, but rarely causes functional impairment [1, 6, 10, 18]. Surgical options in case of PPC with ulnar shortening in patients with partially open physes are resection of the epiphyseal bar, epiphysiodesis of the radial epiphysis, and ulnar distraction osteogenesis. Once skeletal maturity is reached, patients can opt for ulnar distraction or closing wedge osteotomy of the radius [6]. For this reason, it is advised that patients are followed radiographically until skeletal maturity has been reached, as early intervention in case of PPC can lead to less drastic treatment options [1]. This was also the advice we gave to the patient.

## 4. Conclusion

Isolated distal ulnar physeal SH type IV fractures are very rare. They should be treated by anatomic reduction and stabilization to support growth function and restore biomechanics. Most articles report difficult closed reduction, often caused by soft tissue interposition which necessitates conversion to an open technique with stabilization of the fracture.

Follow-up is necessary until skeletal maturity. Patients and their parents should be informed about the possibility of premature closure of the ulnar physis and subsequent deformity. Despite this, they should also be reassured that slight ulnar shortening rarely poses any functional problems and that there are favourable options for secondary surgical intervention if necessary [1, 6].
